# The Socioeconomic Determinants of Health: Economic Growth and Health in the OECD Countries during the Last Three Decades

**DOI:** 10.3390/ijerph110100815

**Published:** 2014-01-08

**Authors:** Guillem López-Casasnovas, Marina Soley-Bori

**Affiliations:** 1 Centre for Research on Health and Economics (CRES), Barcelona, Catalonia 08002, Spain; E-Mail: msoley@bu.edu; 2 Department of Economics, Universitat Pompeu Fabra, Barcelona, Catalonia 08002, Spain; 3 Graduate School of Economics, Ciutadella Campus, Universitat Pompeu Fabra-Barcelona GSE, Barcelona, Catalonia 08002, Spain; 4 Graduate School of Economics, Bellaterra Campus, Universitat Autònoma de Barcelona, Barcelona, Catalonia 08193, Spain; 5 Department of Health Policy and Management, Boston University, Boston, MA 02118, USA

**Keywords:** economic crisis, health inequality, health distribution, income inequality, Human Development Index, intergenerational welfare policies

## Abstract

In times of economic crisis, most countries face the dual challenge of fighting unemployment while restraining social expenditures and closing budget deficits. The spending cuts and lack of employment affect a large number of decisions that have a direct or indirect impact on health. This impact is likely to be unevenly distributed among different groups within the population, and therefore not only health levels may be at risk, but also their distribution. The main purpose of this paper is to explore links between unemployment, economic growth, inequality, and health. We regress a measure of health, the Health Human Development Index (HHDI), against a set of explanatory variables accounting for the countries’ economic performance (GDP growth, unemployment, and income inequality), and some institutional factors related to welfare spending and the nature of the health systems for the past three decades. In addition, we explore the causes for different results obtained using an inequality-adjusted HHDI, *vs*. the unadjusted HHDI. We describe a panel data model, estimated by random effects, for 32 countries from 1980–2010, in five-year intervals. Our conclusion is that the high economic growth observed in the last decades, together with an increase in the levels of income inequality and/or poverty, explain the observed changes of our index, particularly when this indicator is weighted by health inequality. The remaining institutional variables (the share of social spending, health care expenditure, and the type of health systems) show the expected sign but are not statistically significant. A comment on the methodological pitfalls of the approach completes the analysis.

## 1. Introduction

The analysis of the impact of economic growth on health, and the impact of health on economic growth is still a very challenging issue in the health economics literature [1] today. In fact, there exists literature that attempts to model the effects of health on economic growth [2], while another focuses on the reverse: how health changes as a result of economic growth. Although there may be some endogeneity problems in both approaches—particularly in the variable economic growth, since this may be explained by, among other things, the level of a population’s health—we pursue here a further step in the analysis of the effects of growth on health and particularly on health inequality. 

Political scientists often claim that income inequality lowers the health of the poor by frustrating their expectations and capacity for self-fulfillment and instilling a sense of failure. This hypothesis is quite difficult to prove, however, because one cannot compare two countries or individuals with equal income but unequal distributions (e.g., the average income in Ghana and that of low deciles in Harlem) without a proper adjustment of all the remaining parameters. The relevant links may be otherwise tested at the macro level through the dynamics of income in a specific country, or at the micro level by testing whether the poorest today in a place of high income and inequality are in better health than in the past. The relationship between income and health is likely non-lineal and may be unaffected by short-run impacts. Finally, health and income cannot be analyzed as two separate entities under a single causality link. 

The goal of this paper is to explore the links between economic growth, inequality, institutional factors (such as social and health expenditure) and health in the OECD during the last three decades. Health is measured through the Health Human Development Index (HHDI) and also through the inequality-adjusted HHDI. To our knowledge, this is the first paper exploring longitudinally the interplay among these variables in the OECD countries employing step by step hypothesis testing. We improve the specification of existing health models by including not only economic growth and inequality indicators, but also unemployment rate, the existence of a National Health Service, and the magnitude of social and health expenditure. Moreover, the inequality-adjusted HHDI captures better than the unadjusted measures the multidimensional nature of health-related social welfare by measuring not only health status but also its distribution. The paper is organized as follows: Section 2 reviews the existing literature exploring relationships among income, inequality and health. Section 3 describes our empirical model and Section 4 presents our results. Finally, Section 5 provides conclusions and some policy recommendations based on our findings.

## 2. Previous Literature

### 2.1. Theoretical Foundations

Income rises with improving health, and the improvement of health serves multiple goals other than simply income growth [3]. However, achieving this virtuous cycle does not imply an improvement in income redistribution, although these improvements can create a Pareto superior situation. The utilitarian Pigou-Dalton principle (under the Pigou-Dalton principle a transfer of income from a richer to poorer person, so long as that transfer does not reverse the ranking of the two, will result in greater equity. This principle is theoretically consistent in the single dimension setting because regressivity in terms of attribute amounts and regressivity in terms of individual well-being coincides in the case of a single attribute. In the multidimensional setting, however, the relationship between the various attributes and well-being is more complex. To formulate a multidimensional Pigou-Dalton transfer principle, a concept of well-being should therefore first be defined) and the Rawls maxi min rule (according to Rawls, social and economic inequalities are to be arranged so that they are to everyone’s advantage and attached to positions open to all) allow for an increase in welfare without loss of generality, even in a case of increased income inequality. There is no doubt that income loss reduces welfare while gains in health increase it, but determining who benefits and how much is important because social weights are beyond the slope of the welfare function. If health improvements are actually achieved by redistributing a pre-existing income level, we need a welfare function (second theorem of welfare economics) in order to judge those social gains. 

The analysis of the decomposition effect of the interrelationship between income and health goes beyond single links and focuses on the joint income-health distribution. Three main questions arise: (i) How does an increase in income affect health across different health statuses? Or, more precisely, how does greater income increase one’s well-being, especially those in poorer health? (ii) Is one’s well-being concave in relation to income? If so, this would imply that by simply transferring income from wealthier to poorer individuals, social welfare improves (Dalton-Pigou). (iii) Does the income concavity of social welfare decrease with higher health levels and, as a result, in order to improve social welfare, will a transfer involving poor and sick people have a greater impact than one aimed at those who are poor but in good health? 

It is also worth noting that health inequality associated with income is related to mean income through income elasticity. This measures how mean health responds to a proportional increase in income and how this income elasticity varies with income levels. According to some sources [4], Greece and Germany are the most dependent on income elasticity for health coverage among the European countries. In addition, health inequality related to income is influenced by factors other than pure income inequality. For this, the effect of mean income on elasticity needs to be adjusted by factors other than income and the income rank. Income-related inequalities from determinants such as age, for instance, and how they concentrate for social groups, appear to be the most relevant factors. In this sense, Contoyannis and Forster [5] show that a public policy that reduces income inequality may, under certain circumstances, and given the sign of the former effects, leave health inequality unchanged—or even raise it. As such, income elasticity of health inequality may play a crucial role because if it increases with income, a proportional income growth may lead to higher income-related health inequality. Otherwise, if income growth goes hand in hand with a reduction in health inequality, then greater social inclusion derives from both as a windfall profit.

### 2.2. Empirical Studies about Income, Inequality and Health

Van Ourti *et al*. [6] provide some evidence of the effects of income growth and inequality on health inequality in Europe. Indeed, they analyze how income distribution varies as income-related health inequality (IRHI hereafter) changes, disentangling the effect of proportional income growth from the impact of changes in income inequality. This is made by estimating two hypothetical health levels: first, the health level that would prevail in the event of a non-changing income distribution; and second, the health level that would prevail in case of a proportional income growth. This enables isolating the effect of changes in the income distribution from the other health determinants as well as income inequality variations from proportional income growth. In both instances, there is a direct effect of the income redistribution on IRHI (which depends on the slope of the income elasticity), but also an indirect impact, through the other health determinants (which essentially depends on the concentration index of the other health determinants). Evidence suggests that pro-poor changes in income inequality do not always lead to reductions in IRHI if income inequality and elasticity do not move together “on average”. 

For the European countries, Van Kippersluis *et al.* [7] analyze the relationship between health and income across the life cycle of several generations in Europe. They observe in general a moderate and steady decline in mean health until the age of 70, followed by a steep acceleration in the rate of deterioration thereafter. In southern Europe and Ireland, where development has been most rapid, the average health of generations born in more recent decades is significantly higher than that of older generations. This is not observed in the northern countries. Moreover, in almost all countries of EU-11, health is more dispersed among older generations now than in the past, and indicates that, despite this, Europe has experienced a reduction in overall health inequality over time. On the whole, health inequality does not seem to increase as a given cohort ages. Finally, there is no overwhelming evidence that IRHI is greater among younger generations than older. Indeed, in some countries, including the United States, the income gradient in health does peak around retirement age.

For Sweden, Islam *et al*. [8] analyze precisely how aging may impact income-related health inequality. If health inequality increases as the population ages, then the question is whether aging generates inevitable inequality consequences not amenable to public policy interventions. These authors conclude that good health is generally pro-rich, and this bias increases as the cohorts become older. The age-gender standardization does not avert this trend. The increasing trend in health inequality is then partly explained by the decrease in the population mean of health, which is attributable to the aging population. It is also well known that the variation of health of different cohorts increases over time. To be precise, elderly people in lower health states remain in the poor group, which then drives the inequality upwards. Conversely, the “student effect” or “young effect” biases the index downwards, since young people are, on average, healthy but poor. No evidence suggests that health profiles across individual-mean income groups diverge over time, however the observed increase in income-related health inequality may be an artifact related to the structure of the pension payments system (the “retirement effect”) or to changes in the saving behavior at older ages. By using lifetime income data, the authors find that the concentration index is quite stable over time. Indeed, due to this influence on redistribution, the ranking of the individuals at a given moment is strongly influenced by the pension payments. In Sweden, however, when one controls for age-related income mobility over the life cycle, there is little evidence that income-related health inequality increases as the population ages.

Finally, for the United States, Deaton and Paxson [9] found that health deteriorates with age at a persistent, constant rate, and that health variance increases up to the age of 60, after which it remains constant. In addition, they argue, if we assume that shocks are cumulative and not random, the prediction of increasing variance with age would no longer hold. These authors also found that the health income gradient is greater among young cohorts, and that the socioeconomic components of inequality in health have been rising, while total health inequality, measured by the variance, has been falling. 

Existing research directly assessing the relationship between income inequality and health presents mixed results. Cross-sectional studies (e.g. [10], [11]) mostly support a negative relationship between income inequality and health. However, they are based on oversimplified health models that disregard country specific factors such national policies to improve health and health services. Very few studies have explored this research question using time series data. Mellor & Milyo [12] analyze the relationship between health, the Gini coefficient, GDP *per capita* and school enrollment for a sample of OECD and non-OECD countries. Health is measured through life expectancy at birth and infant mortality. Their cross-sectional analysis suggests that the Gini coefficient is positively related to life expectancy and negatively associated to infant mortality. However, when the model is adjusted for GDP and school enrollment, the statistically significant effect of the Gini coefficient vanishes. Their longitudinal analysis includes data from 1960 to 1990 in 10 years intervals and estimates first difference models, considering 10 and 20 years change. Both models do not support the hypothesis that inequality has a negative impact on health. As part of their sensitivity analysis, they constrain their study sample to 12 OECD countries between 1960 and 1990. The pooled cross sectional analysis does not find a significant relationship between inequality and health.

To sum up, research exploring the interplay among income, inequality and health suggests: (i) the relationship between income inequality and income related health inequality is not direct [6], (ii) health inequality does not depend on age [7], (iii) higher income generally contributes to better health but there are some exceptions (such as the case of Sweden [8]), (iv) health inequality related to socioeconomic factors has increased over time but total health inequality has decreased [9], (v) based on cross-sectional studies, income inequality has a negative impact on health, however, longitudinal research does not support such an association. Our study tries to contribute to this discussion by exploring both inequality adjusted and unadjusted health models on a longitudinal and cross-sectional fashion for 32 OECD countries from 1980–2010. Specifically, we believe that our model contributes to existing research testing the contribution of unexplored health determinants such as unemployment rate, social expenditure, health expenditure and the existence of National Health Services. Also, we test several models for health adjusted for inequality and estimate which elements drive observed differences with unadjusted model. 

## 3. Empirical Model

In analyzing the observed relationships between economic performance and health and social welfare, we limit our analysis at the aggregate level. We take as a proxy for welfare the health component of the United Nations Human Development Index during the last thirty years. In order to test the impact of economic growth, unemployment, and income inequality on health, we will take advantage of the new data on the health index variation, once the HHDI is weighted by observed inequality. The idea of weighting health status indicators by inequality in trying to capture welfare derived from health is sound, as has been extensively argued in the previous section. In fact, welfare from health is not just related to the size of the index, but also to its distribution. Both factors indeed influence social welfare. 

In this sense, the new Human Development Index (a description can be found in [13]) has recently tried to capture the impact of inequality not just for health, but for any of the components of the Human Development Index (health, education, and material welfare). Inequality in health in the HDI report is measured in terms of the variation between the 5-year cohorts in life expectancy at birth, as collected by the United Nations Department of Economics and Social Affairs. Life expectancy here is defined as the number of years a newborn infant could expect to live if prevailing patterns of age and specific mortality rates at the time of birth were to remain constant throughout the infant’s life. Data are taken from the abridged life table across age intervals of five years, with the mortality rates and average age of death specified for each interval. As an element of the general HDI, the health inequality component is taken for aggregation as a part of a geometric mean. Its dimension is defined as the difference between the actual (observed) and the minimum value of life expectancy, divided by the difference between the observed maximum value and the minimum (established at 20 years). 

Among the OECD countries, to account for the inequality, adjusted index means an average drop of 5% with regard to the unadjusted one (a 38% drop for Less Developed Countries, or LDCs). However, in comparing health and income with and without inequality weighting, we observe a lower drop for LDCs (and a higher figure for the developed countries) than that of the *per capita* income (weighted *vs*. un-weighted, [14]). By comparing the index with and without inequality weights, our analysis aims to understand which explanatory factors move health inequality toward lower index values. 

Premises for this exercise, according to the analysis of the previous section, are: (i) the trend in the economic performance during a range of years previous to the period analyzed affects health, since it may be expected that greater income growth is associated with greater stress in an uneven (social gradient) manner. This means that countries with a higher average economic growth may impel their working populations toward situations that reflect higher health index inequality; (ii) changes in employment status affect the health index, as redundancies create among the population a sense of precariousness, depression, and lack of self-esteem that may affect the deterioration of the indicator; (iii) finally, as argued earlier, the level of poverty and the income inequality of the countries as measured by the Gini coefficient may be expected to lower the health index, once weighted by inequality.

In understanding the role of inequality in the observed health levels [15], we must remember that health is a difficult domain in itself, either on a theoretical or empirical basis, particularly if we are concerned with the measure of inequality of achievement. Child mortality data, often used to represent health inequality in developing countries, are not always available, nor is there an alternative international indicator of general health. Life expectancy data are commonly used in aggregate indicators but are not available at the individual level or by population subgroups in all countries. At any rate, relying on data from life tables, it is possible to estimate a lower bound of inequality by constructing the means of the distribution of life expectancy for different age cohorts of the population. Of course, this measure of *between-group* inequality is only as accurate as the tables from which it is drawn. This measure also smoothes inequality within each age cohort, and does not take into account disability or morbidity, but only presence of physical life. Given the absence of other data sources with sufficient coverage across countries, however, this seems to be the approach that will generate the most realistic approximation to health inequality. Thus, in pursuing health equality without controlling for life expectancy at birth across different generations (cohorts, age groups), progress in health might be viewed as undesirable should this coincide with an increase in inequality. Otherwise, we should implicitly accept that society is willing to compensate with additional benefits those who were born “by accident” in early generations. This is a rather controversial issue in and of itself.

Despite those difficulties, and having considered the previously stated aspects, we proceed to present the central part of our empirical study in estimating the links between health and a country’s economic performance, including wealth generation and income distribution. Our sample consists of 9 socioeconomic indicators for 32 OECD countries (Australia, Austria, Belgium, Canada, Chile, Czech Republic, Denmark, Finland, France, Germany, Greece, Hungary, Iceland, Ireland, Israel, Italy, Japan, Korea, Luxembourg, Mexico, Netherlands, New Zealand, Norway, Poland, Portugal, Slovak Republic, Spain, Sweden, Switzerland, Turkey, United Kingdom, and the United States of America), including: the inequality-adjusted and unadjusted health indices, the growth rate of the GDP *per capita*, the unemployment rate, the inequality in the distribution of wealth (measured by the Gini coefficient), the poverty rate, and three dummy variables capturing the level of social and health expenditure and the existence of a National Health System. 

All the data was obtained from publicly available data sources including the OECD, World Bank and the United Nations Development Program websites. 

To estimate this model, we use random effects. As it is known, fixed effects are more appropriate when the purpose is to control for omitted variables, assuming they are constant over time. Hence, this methodology does not allow including variables that characterize the countries, such as the type of health systems they have. On the other hand, the estimation by random effects allows regressors to be invariant over time and requires that the country-specific effect is independent of the explanatory variables, which we believe is the case in our sample. Most of our variables are different across countries but relatively constant over time, such as the social expenditure, the health expenditure, and the existence of a National Health System.

At any rate, once random effects are validated against fixed effects, the Hausman test (the null hypothesis of the Hausman test is that fixed effects and random effects do not differ substantially and, thus, random effects should be employed because it is more efficient than fixed effects (it takes into account both within and between country variability). In our case, the *p*-value = 0.62. Thus, we fail to reject the null hypothesis and, accordingly, choose random effects) allows us to accept the hypotheses that this is not biasing our results. 

## 4. Results

Table 1 presents descriptive statistics for our main study variables across time. The Health Index constantly improved over time, from 0.83 in 1980 to 0.93 in 2010. GDP growth was relatively stable despite an abrupt reduction in 2005, from 4.82% in 2000 to −3.39% in 2005. The unemployment rate steadily increased since 2000 up to 8.40% in 2010. Finally, the Gini coefficient (displayed in a 0–100 scale) showed a slight increase across time, which implies more inequality in the distribution of income.

**Table 1:** Descriptive statistics for the main study variables.

	1980	1985	1990	1995	2000	2005	2010
**Health Index**							
N	32	32	32	32	32	32	32
Mean	0.83	0.85	0.86	0.88	0.90	0.92	0.93
Std. Dev.	(0.06)	(0.05)	(0.05)	(0.06)	(0.05)	(0.05)	(0.05)
**GDP Growth Rate**							
N	28	28	29	32	32	32	32
Mean	2.31	3.42	3.47	3.44	4.82	−3.39	2.48
Std. Dev.	(2.53)	(1.42)	(2.77)	(3.18)	(2.05)	(2.49)	(2.33)
**Unemployment Rate**							
N	25	25	26	30	32	32	32
Mean	4.60	6.99	5.90	8.04	6.64	6.89	8.40
Std. Dev.	(2.71)	(4.32)	(3.12)	(3.94)	(3.41)	(3.01)	(3.81)
**Gini Coefficient**							
N	0	25	13	25	27	28	32
Mean		29.11	31.52	30.98	31.89	31.22	32.31
Std. Dev.		(6.42)	(8.58)	(7.41)	(7.51)	(5.70)	(6.85)

We begin by approaching a model in which the health index is explained by the economic performance (GDP *per capita* growth rate), the unemployment rate of the period, and income inequality (the Gini coefficient). As model 1 on Table 2 shows, higher average economic growth and higher observed Gini coefficients make for lower levels of health. Hence, it seems to confirm that economic growth has a cost in terms of more social stress and lower health indexes, as happens with income inequality. Neither the change in the unemployment rate for the period nor the average value are statistically significant, despite having the expected sign (higher unemployment, lower health indexes; the idea that unemployment is good for health can be found in [16]. However, this may be a pure short-run effect (more jogging and higher caloric intake). More of the public health studies derive a negative sign given the associated factors to unemployment, such as income reduction and depression). Notice how the value of the constant is significantly close to 100 (the health index takes values between 0 and 1, where the closer it is to 1, the better the health level of the populations. We adjust the index to a 0–100 scale by multiplying it by 100 for practical purposes. This modification does not alter the essence of our results), the maximum value of the health index, illustrating the high development of most of the OECD countries.

**Table 2:** Longitudinal results: 1980–2010-Health Index as the dependent variable.

Controls	Model 1		Model 2		Model 3		Model 4	
Constant	Estimate	95% CI	Estimate	95% CI	Estimate	95% CI	Estimate	95% CI
	92.53 *** (3.5)	(85.67, 99.39)	93.44 *** (4.01)	(85.60, 101.29)	87.60 *** (4.21)	(79.35, 95.86)	87.56 *** (4.23)	(79.25, 95.87)
GDP *per capita* growth rate	−0.286 ** (0.07)	(−0.43, −0.14)	−0.287 ** (0.07)	(−0.43, −0.14)	−0.268 ** (0.07)	(−0.42, −0.12)	−0.269 ** (0.075)	(−0.42, −0.12)
Unemployment rate	−0.122 (0.12)	(−0.36, 0.12)	−0.119 (0.13)	(−0.37, 0.13)	−0.108 (0.12)	(−0.35, 0.14)	−0.105 (0.13)	(−0.35, 0.15)
Gini coefficient	−0.196 * (0.11)	(−0.41, −0.02)	−0.213 * (0.11)	(−0.441, 0.15)	−0.105 (0.12)	(−0.33, 0.12)	−0.095 (0.12)	(−0.32, 0.13)
Social expenditure			−0.37 (1.58)	(−3.46, 2.73)				
National health service			−0.42 (1.50)	(−3.37, 2.52)			−0.722 (1.47)	(−3.61, 2.16)
Health expenditure					3.79 * (1.48)	(0.88, 6.71)	3.89 * (1.55)	(0.86, 6.93)
N	136		136		136		136	
Wald Chi^2^	15.58		15.98		23.22		22.81	
Prob > Chi^2^	0.0014		0.007		0.0001		0.0004	
Overall R²	0.240		0.249		0.276		0.277	
Between R²	0.422		0.445		0.387		0.379	

Notes: The robust standard deviations are presented in parenthesis beside the estimated coefficient. Significance levels: * *p* < 0.05, ** *p* < 0.01, *** *p* < 0.001.

To ensure that these results are robust to some potentially important omitted variables, we include two dummies in our estimation. The first one tries to collect the different role of social expenditure for each country throughout the period. We consider a value of 20% as the minimum proportion of social spending in GDP to act as a buffer for all those external fluctuations in the explanatory variables, which can reduce the impact of the remaining variables on our health index. In other words, all other things equal, a relatively important welfare state may mitigate some of the otherwise negative effects on health. Another dummy deals with the different nature of the health system under consideration; more precisely, it takes a value of 1 for those countries with a National Health Service (NHS) model, and a value of 0 for those with a social health insurance system. The hypothesis is that more state-administered systems, as the main characteristics of a NHS, should be better equipped to lower the impact of the remaining explanatory variables on health.

As the results of model 2 suggest, the nature of the health system does not alter the sign of the effects of the other variables on the health index. GDP and the Gini index maintain the expected statistically significant impacts and, despite the negative value of both dummy variables, they are not statistically significant.

We finally explore the role of health care expenditure over GDP, in isolation and together with the nature of the health systems (NHS), for each country in our series. For this purpose, we create a new dummy variable that takes the value of 1 if the OECD country has a health expenditure equal to or greater than 7.5% of the GDP (around the average of the OECD health expenditure), or a value of 0 if this indicator is lower than 7.5%. The results (models 3 and 4) show a positive and significant effect of health spending (higher expenditure, higher health index), together with a reduction (not statistically significant) on the impact of inequality. The unemployment rate remains non-significant. Notice how both models 3 and 4 improve the overall R-squared (from 0.249 to 0.277). 

In the second part of our analysis, we consider the health index adjusted for inequality, on a cross-section basis, just for the year 2010—the only year for which this index is available at present. Despite the small sample size, we regress the above-mentioned vector of explanatory factors (economic growth, income inequality, and unemployment) during the period 2000–2010, aiming to understand the inertia of the main driving forces of the health index once this is adjusted for inequality. As model 5 shows (Table 3), higher average economic growth and higher observed Gini coefficients are associated with lower levels of the inequality adjusted health index. Therefore, it seems to confirm that economic growth has a cost in terms of welfare. Neither the change in the unemployment rate for the period nor the average show statistically significant parameters, although they hold the expected sign (higher unemployment, lower health indexes); most likely this is due to the correlation between economic growth and unemployment (−0.38). 

This result also holds when we include the poverty rate (the child poverty index; see Model 9 in the Appendix, where we use “poverty among working adults” as an explanatory variable. According to our results, neither this variable nor the unemployment rate seem to be significant variables in explaining the health index) rather than the Gini coefficient (model 6). There is a positive high correlation index (0.864) between poverty rate among children and income inequality. We obtain negative and significant coefficients for both average economic growth and child poverty, which suggests that higher levels of these variables are bad for the health index, as maintained by most of the literature previously reviewed. 

We finally explore variables related to the change in the health index once we weight it by inequality; thus, we try in these final regressions to estimate the explanatory factors behind the difference between the conventional health index and the health index adjusted for inequality as calculated in the new HDI. 

We can show from the regression results (model 7) that, in absolute terms, the drop of the ∆HI (the Health Index when it is adjusted for inequality) is basically explained by economic variables, particularly economic growth and, with less statistical significance, by the unemployment rate. Evidence suggests that higher economic growth leads to a greater difference between weighted and un-weighted values. The Gini coefficient seems to explain the larger part of the variation: a higher income inequality means a higher change between the adjusted and unadjusted index. Notice the high F-test value for the latter specification, which reinforces the significant coefficients obtained. In similar terms (for the remaining variations of this model, see Appendix, models 10 to 13 where we analyze how these results change if, instead of considering the percentage change, we use the average value of the independent variables for the period) we confirm the former result by taking the % rather than the absolute value of the drop of the index (HIL) (model 8). We observe that, again, higher unemployment rates, greater Gini Index, and greater economic growth lead to a larger percentage fall in our health index, after we adjust it for inequality. Economic growth and the Gini Index have a statistically significant effect, however, the unemployment rate is non-significant. Notice how the adjusted R-squared suggests that these three explanatory variables, once inequality is considered, account for more than 40% of the variation in the percentage loss in the health index.

**Table 3:** Cross-sectional results: 2010-Health Index adjusted for Inequality (HII).

	Model 5		Model 6		Model 7		Model 8	
**Dependent Variable**	HHI		HHI		ΔHI		HIL	
**Controls**	Estimate	95% CI	Estimate	95% CI	Estimate	95% CI	Estimate	95% CI
Constant	98.5 *** (41.6)	(90, 107.1)	98.9 *** (2.60)	(93.6 , 104.3)	−0.26 *** (1.33)	(−2.96, 2.46)	−80.2 *** (171.1)	(−430.6, 270.2)
GDP *per capita* growth rate, % change 2000–2010	−0.03 * (0.01)	(−0.06, −0.006)			0.012 ** (0.005)	(0.0025, 0.24)	1.50 * (0.60)	(0.30, 2.70)
Unemployment rate, % change 2000–2010	−0.02 (0.01)	(−0.04, 0.01)			0.007 (0.004)	(−0.0012, 0.02)	0.93 (0.54)	(−0.19, 2.04)
Gini coefficient 2010	−31.4 * (12.5)	(−57.1, −5.7)			17.0 *** (4.0)	(8.86, 25.3)	2012 ** (515)	(956.6, 3,066)
Average GDP *per capita* growth rate, 2000–2010			−1.9 * (0.80)	(−3.70, −0.30)				
Poverty rate among children 2005			−0.41 * (0.160)	(−0.73, −0.07)				
N	32		30		32		32	
F	4.71		7.69		9.53		8.36	
Prob > F	0.0088		0.0023		0.0002		0.0004	
R²	0.335		0.363		0.505		0.472	
Adjusted R²	0.264		0.316		0.452		0.42	

Notes: (1) The robust standard deviations are presented in parenthesis beside the estimated coefficient. (2) Significance levels: * *p* < 0.05, ** *p* < 0.01, *** *p* < 0.001. (3) ΔHI = difference between the traditional health index and the health index adjusted for inequality. HIL = percent loss in the health index when it is adjusted for inequality.

Finally, we introduce age as an explanatory variable in models 7 and 8. The underlying hypothesis is that the difference between the un-weighted and the weighted health index (rather than its absolute value, essentially approached by life expectancy), should be sensitive to differences in the share of the aged population of the countries. This might be the case due to the fact that increasing depreciation rates of health at older ages, *within* each country, makes population to converge towards relatively worse health states with a more equal distribution, since that depreciation is less dependent on income inequality. This larger share of the aged population would partially offset any other values of health inequalities for the rest of the age cohorts. In our sample, the introduction of the share of the population over 65 years old does not prove to be statistically significant. This is the case (see Supplementary Table S1, model 14 and 15) when we either regress age on the total level of the inequality-adjusted health index, or rather on its drop with regard to the unadjusted level.

In summary, the results of our empirical exercise seem to confirm a significant negative influence of economic growth, income inequality, and poverty on the health index adjusted for inequality. Indeed, economic growth and income inequality appear to mostly explain the variation of the difference between the traditional health index and the one adjusted for inequality.

## 5. Conclusions and Further Research

The results from the panel data analysis including 32 OECD countries from 1980–2010 suggest that the GDP *per capita* growth rate, the Gini Index and health expenditure have a consistent significant relationship with the Health Index. Specifically, economic growth and income inequality have a negative effect on the Health Index, while health expenditure seems to contribute to better health. The results from the cross sectional analysis using the inequality-adjusted Health Index as the dependent variable confirmed the panel data results and showed a negative impact of the poverty rate on health. Finally, we also found that the difference between the traditional Health Index and the one adjusted for inequality is mostly driven by economic growth and income inequality.

Given what we now know, despite the observed relationship between income variations and health, more analysis is needed on the epidemiology and macroeconomics of social factors when we move from individual to collective aggregate behavior of income and health. This requires improving modeling at the individual level, such as for rational behavior, or delaying the entrance into addictive consumption. We need better empirical approaches to deal with the endogenous relationship between health and income by instrumenting adequate variables. The endogeneity is mostly due to the existence of cumulative causation processes related to social conditioning, labor employment/capital input technologies, and factor interactions. At the aggregate level, we need to focus on the epidemiology and macro social determinants of health, such as those derived by the intergenerational impact on equity (the welfare of children: cognitive and non-cognitive human capital accumulation). Furthermore, we should be more aware of the consequences of taxation practices in financing public spending (dual fiscal systems presently lead to regressivity), tax wedges on residual income, the education gap (and its effects on smoking and obesity, among others), and of zoning laws and housing prices (due to the mobility and obesity effects). Corporate practices and nutrition habits (production and design, marketing, retail distribution, and pricing) may be influential too. 

In our estimation, some methodological pitfalls remain. First, we need structural models rather than reduced form equations and longitudinal-panel data analysis since health policy is concerned with lifetime analysis. For instance, Eurostat (see Methodological issues in the analysis of the social determinants of health using UE-SILC data [17]) follows this methodology to determine how income-related health inequalities change in short- and long-term perspectives, using an index of health-related income mobility based on a concentration index (CI; The concentration index provides a summary measure of the magnitude of socioeconomic-related inequality in a health variable of interest. It is the result of multiplying by 2 the area between the concentration curve and the 45º line. The two key variables underlying the concentration curve are: the health variable, of which the distribution is the subject of interest; and a variable capturing living standards, against which the distribution is to be assessed). For the European member states, results indicate the existence of long term income-related inequalities in health, and low-income individuals seem more vulnerable to suffer health limitations. Moreover, the values of the mobility proxies suggest that income dynamics should be taken into account when inequalities are computed in health limitations in order to avoid overestimation. Aware of the drawbacks of the CI (first, the bounds of the CI depend on the mean of the health variable. Second, different rankings are obtained when comparing inequalities in health with inequalities in poor health [18]). Third, the index becomes arbitrary if qualitative health variables are used), in a further step, the previously-mentioned EU report uses the “adjusted” CI [19] to compare groups with a different average health.

Second, publicly available individual and community-level datasets are required to verify population level findings like those presented in our study. Individual-level data allows exploring the determinants of individuals’ rather than population’s health. Therefore, it is better equipped to inform health policy. Also, it facilitates a more refined inquiry into the health-income relationship. Unlike population-level data, we can differentiate and specifically test the absolute-income hypothesis (absolute income influences health), the relative income hypothesis (relative income affects health), the relative-position hypothesis (not only income levels but one’s position in the income distribution matters), and the deprivation hypothesis (income deprivation determines health) [20].

Third, further attention should be paid to non-linearities, adjustment costs, accounting for time in habit formation, unobserved heterogeneity, and changing conditions overtime. Identifying substitution effects is essential, as is the impact of exogenous shocks, technological change, the production model, and more stress and lack of exercise. Equally important are positive complementarities, such as unemployment, spare time, and self-care, and negative complementarities, including unintended effects on the less wealthy, the less educated, and the more addicted. Also, it is necessary to distinguish the sign and direction of the relationships, such as: unemployment and alcohol consumption; alcohol abuse and risky sex; loss of income and higher caloric intake; budget restraint effects on higher tobacco prices; and less expenditure on healthful nutrition. The result of all this may be a dangerous causative accumulation: poorer, less educated, and more addicted. We must therefore disentangle the recursive process for better evidence-based health policies on income and health.

## Supplementary Files

Supplementary File 1 Supplementary Information (PDF, 119 KB)
