# Supplementary material for: The Socioeconomic Determinants of Health: Economic Growth and Health in the OECD Countries during the Last Three Decades
Source: Int J Environ Res Public Health. 2014 Jan 8;11(1):815–29. doi: 10.3390/ijerph110100815 (PMC3924476; doi:10.3390/ijerph110100815)
Supplement: Supplementary File 1 — Supplementary Information (PDF, 119 KB) [file ijerph-11-00815-s001.pdf]

The Socioeconomic Determinants of Health: Economic Growth and Health in the OECD Countries during the Last Three Decades

Table S1. Additional cross-sectional models tested (2010).

	**Model 9**		**Model 10**		**Model 11**		**Model 12**	
**Dependent Variable**	**HII**		**HIL**		**HIL**		**HIL**	
**Controls**	**Estimate**	**95% CI**	**Estimate**	**95% CI**	**Estimate**	**95% CI**	**Estimate**	**95% CI**
Constant	100.4 ***	(93, 100.7)	312.4 ***	(81.6, 543.4)	315.8 ***	(51.5, 580)	−229 ***	(−623.2, 165.1)
Average GDP *per capita* growth rate, 2000–2010	−1.8	(−3.7, 0.1)	83.1 *	(0.18, 166.1)	70.91	(−18.8 , 160.6)	45.7	(−3.22, 123.7)
Average unemployment rate, 2000–2010	−0.41	(−1.0, 0.24)			7.55	(−2.46, 39.7)	3.97	(−23.6, 31.53)
Poverty rate among working age (2005)	−0.44	(−1.1, 0.23)						
Unemployment rate, percentage change 2000–2010			0.63	(−0.67, 1.93)				
Gini index							1,971 **	(801.6, 3,140.4)
N	30		32		32		32	
F	4.04		2.39		1.97		5.78	
Prob > F	0.017		0.109		0.158		0.003	
R^2	0.318		0.142		0.119		0.382	
Adjusted R^2	0.239		0.08		0.058		0.316	

Table S1. *Cont.*

	Model 13		Model 14		Model 15	
**Dependent Variable**	**HIL**		**HII**		**ΔHI**	
**Controls**	**Estimate**	**95% CI**	**Estimate**	**95% CI**	**Estimate**	**95% CI**
Constant	565.7 (53.4) ***	(456.5, 675)	94.78 ***	(73.41, 116.1)	0.455 ***	(−8.07, 8.98)
GDP per capita growth rate, % change 2000–2010	1.76 *	(0.28, 32.3)	−0.035 *	(−0.066, −0.004)	0.012 *	(0.001, 0.02)
Unemployment rate, % change 2000–2010	1.10	(−0.26, 2.5)	−0.015	(−0.043, 0.013)	0.007	(−0.005, 0.02)
Gini index			−26.59	(−68.82, 15.63)	16.22	(−2.45, 34.89)
Elderly population as a % of total population, 2010			0.14	(−0.588, 0.87)	0.027	(−0.29, 0.24)
N	32		32		32	
F (Prob > F)	3.29 (0.051)		3.37 (0.023)		5.98 (0.0014)	
R^2	0.185		0.339		0.469	
Adjusted R^2	0.129		0.235		0.391	

Notes: (1) The robust standard deviations are presented in parenthesis beside the estimated coefficient. (2) Significance levels: * $p < 0.05$, ** $p < 0.01$, *** $p < 0.001$.

© 2014 by the authors; licensee MDPI, Basel, Switzerland. This article is an open access article distributed under the terms and conditions of the Creative Commons Attribution license (http://creativecommons.org/licenses/by/3.0/).
